# Two neurocognitive domains identified for patients with myalgic encephalomyelitis/chronic fatigue syndrome and post-acute sequelae of COVID-19

**DOI:** 10.3389/fneur.2025.1612548

**Published:** 2025-07-15

**Authors:** Ariadna Sandoval, Mingqi Li, Leonard A. Jason

**Affiliations:** Center for Community Research, DePaul University, Chicago, IL, United States

**Keywords:** neurocognitive, hypersensitivity, salience network, myalgic encephalomyelitis, PASC/long COVID, factor analyses, brain fog, default mode network

## Abstract

Patients with Myalgic Encephalomyelitis/Chronic Fatigue Syndrome (ME/CFS) and Post-Acute Sequelae of COVID-19 (PASC) often have neurocognitive complaints that involve memory and concentration problems and difficulties paying attention. Other neurocognitive domains such as hypersensitivity to noise and light have rarely been included as aspects of neurocognitive impairment for these post-viral conditions. The current study evaluated a more extensive list of neurocognitive items for a group of 2,313 patients with ME/CFS and 299 patients with PASC. Exploratory factor analyses found two factors for each patient group, one involving classic memory and concentration symptoms and the other involving sensory overload phenomena. The findings suggest that researchers might consider expanding the types of self-report neurocognitive symptoms among patients with these post-viral illnesses.

## Introduction

Neurocognitive impairment that results in difficulty remembering, concentrating, and making decisions is a central feature of Myalgic Encephalomyelitis (ME/CFS) (1). In a systematic review and meta-analysis by Aoun Sebaiti et al. (2), those with ME/CFS had impairment in visuo-spatial immediate memory, reading speed, graphics gesture, episodic verbal memory (storage, retrieval, recognition), visual memory (recovery), and a low efficiency in attentional abilities, but executive functions were not affected.

Many who have been diagnosed with Post-Acute Sequelae of COVID-19 (PASC) also exhibit neurocognitive problems (3), manifested as impairment in domains such as memory, language, orientation, application, attention, perception (visual, auditory, sensory), and executive dysfunction (4, 5), inattention, aphasia, amnesia (6), word fluency, working speed, delayed recall and attention (7). Structural and functional changes in their brains have been found to correlate with neurocognitive examination scores (8, 9). Cerebrovascular reactivity, a measure of neurovascular function has been associated with cognitive function with extreme values suggesting a dysregulated neurovascular response (10).

In both ME/CFS and PASC, there has been a reported reduction in cerebral blood flow, which may contribute to symptoms of fatigue and cognition (11). Vernon et al. (12), for example, found neurocognitive and symptomatic abnormalities in patients with PASC and ME/CFS. A cardiopulmonary exercise test revealed “preload failure” in patients with ME/CFS and PASC (13). This dysfunction refers to the impaired ability to deliver sufficient blood to the heart for it to adequately fill, resulting in a reduced cardiac output and a decrease in the amount of blood supplied to the muscles during exercise.

Two ME/CFS case definitions provide a more expanded list of neurocognitive symptoms. The Canadian Consensus Criteria definition ME/CFS (14) requires at least two of the following symptoms: impairment of concentration and short-term memory; difficulty with information processing, categorizing and word retrieval; perceptual and sensory disturbances (e.g., spatial instability and disorientation, and inability to focus vision); disorientation; motor disturbances (e.g., muscle twitches, loss of balance and clumsiness), and overload phenomena: cognitive, sensory (e.g., hypersensitivity to noise and light). In addition, the ME/CFS International Consensus Criteria (15) requires at least one neurological symptom from 3 out of 4 categories: pain, sleep disturbances, neurocognitive impairments, and neurosensory, perceptual, and motor disturbances. Within the neurocognitive impairment category, symptoms include difficulty processing information (e.g., impaired concentration, difficulty making decisions, cognitive overload) and short-term memory loss (e.g., difficulty remembering what one wanted to say, word retrieval, and recalling information). Symptoms in the neurosensory, perceptual, and motor disturbances include an inability to focus, impaired depth perception, and sensitivities to lights, noises, smells, etc., and as well as poor coordination, feeling unsteady on feet, and muscle weakness and twitching.

Based on the Canadian Case Criteria (14) and ME/CFS International Consensus Criteria (15), impairment among those with ME/CFS might affect domains that have been less often studied, including spatial instability, disorientation, and overload phenomena such as hypersensitivity to noise and light. For example, Sharetts et al. (16) found that taste dysfunction was in nearly one-third of individuals with COVID-19, and Maeda et al. (17) found that hypersensitivities were common among patients with ME/CFS. In our study, we examined these symptoms along with more classic neurocognitive symptoms reported for those with ME/CFS and PASC to determine whether they are aspects of the neurocognitive impairment reported by patients with these post-viral conditions.

## Methods

### Participants

#### ME/CFS samples

The dataset for the current study was aggregated across a variety of international samples and included 2,313 patients with ME/CFS and 299 patients with PASC. The sociodemographic information for this sample is reported in Table 1.

**Table 1:** Sociodemographic information for the samples with ME/CFS and long-COVID.

Demographic	ME/CFS Sample (*N* = 2,313)	Long-COVID Sample (*N* = 299)
	***M* (*SD*)**	***M* (*SD*)**
Age (years)	46.93 (13.66)	44.05 (12.88)
Gender		
**% (*n*)**	**% (*n*)**
Female	81.0 (1873)	81.6 (244)
Male	17.6 (407)	15.7 (47)
Other	0.1 (2)	1.7 (5)
Race		
White/Caucasian	76.4 (1766)	90.3 (270)
Asian or Pacific Islander	5.7 (132)	3.3 (10)
Black/African American	0.1 (2)	1.7 (5)
American Indian or Alaskan Native	0.1 (2)	2.3 (7)
Other	1.1 (26)	7.0 (21)
Latinx	5.8 (135)	7.4 (22)
Marital status		
Married	53.4 (1236)	59.9 (179)
Never married	25.9 (598)	99.7 (74)
Divorced	13.0 (301)	11.4 (34)
Separated	4.6 (106)	2.7 (8)
Widowed	1.5 (35)	1.0 (3)
Work status		
On Disability	20.8 (480)	9.7 (29)
Unemployed	6.7 (154)	10.7 (32)
Retired	5.4 (124)	5.0 (15)
Working full-time	3.4 (79)	53.2 (159)
Working part-time	3.0 (69)	15.4 (46)
Homemaker	2.4 (55)	7.4 (22)
Student	1.1 (25)	6.0 (18)

##### DePaul sample

DePaul University recruited an international convenience sample of adults who self-identified as having ME/CFS. Eligibility criteria included being at least 18 years of age, having a self-reported and current diagnosis of CFS or ME, and the ability to read and write in English. There was a total of 217 adults with available data, but three were excluded due to incomplete data. This present study included a total of 214 participants who were predominantly female (84/0%), with a mean age of 52.0 years (*SD* = 11.3), and the majority (74.7%) completed at least a standard college degree.

##### BioBank sample

The BioBank sample was collected by the Solve ME/CFS Initiative^1^. Five hundred and eight participants were recruited by a physician and had been previously diagnosed with ME/CFS. Eight participants were excluded from this present study due to missing data. Of the 500 total participants in the sample, 76.8% were female, with the mean age of 54.8 years (*SD* = 12.0), and 69.9% had completed at least a standard college degree.

##### Newcastle sample

Due to a suspected diagnosis of CFS, all participants in this sample were referred to the Newcastle-upon-Tyne Royal Victoria Infirmary clinic in Great Britain. An experienced physician conducted a comprehensive examination and medical history. Due to incomplete data, five participants were excluded. With a majority of the 95 participants used in this current study being female (82.1%), 50.0% obtained at least a standard college degree and the mean age was 45.8 years (*SD* = 14.1) and.

##### Norway 1 sample

Participants were recruited from southern Norway and contacted via healthcare professionals, ME/CFS patient organizations, and the waiting list for a patient education program. Individuals living with ME/CFS were invited to participate in a randomized controlled trial for a ME/CFS self-management program. To be eligible, participants were required to have a diagnosis of ME/CFS by a physician or medical specialist, be at least 18 years of age, and be physically able to attend the self-management program. Those who decided to participate were confirmed to have a ME/CFS diagnosis. There was a total of 176 participants and 173 included in the present study (3 participants had incomplete data). Approximately half the sample (50.3%) completed at least a standard college degree. The sample had a mean age of 43.3 years (*SD* = 11.7) with the majority of the sample being female (87.2%).

##### Norway 2 sample

Participants were recruited from an outpatient clinic at a multidisciplinary ME/CFS center and from an inpatient medical ward for severely ill patients. Patients were required to be able to read and write in Norwegian and to be between 18 and 65 years of age. All participants suspected of a diagnosis of ME/CFS participated in comprehensive medical examination and history conducted by an experienced psychologist and physician. Due to missing data, 60 of the 64 original participants were included in the present study. Less than half the sample (38.3%) had completed at least a standard college degree. The sample was 81.7% female, with a mean age of 35.4 years (*SD* = 11.7).

##### Norway 3 sample

All participants, recruited from a tertiary care center specializing in ME/CFS, were examined by an experienced physician and determined to meet ME/CFS criteria. Eligibility criteria included being between 18 and 65 years of age and being able to read and write in Norwegian. There was a total of 175 participants, but the current study included 169 with six being excluded due to incomplete data. The majority were female (81.7%), more than half (57.4%) received at least a standard college degree and the mean age was 38.6 years (*SD* = 11.2).

##### Chronic illness sample

A convenience sample of adults living with chronic illnesses was collected by DePaul University as a part of a larger study (18). Participants were recruited online via social media platforms, support groups, and research forums. There was a total of 441 participants who reported an ME/CFS diagnosis. Due to incomplete date, 6 participants were excluded: bringing the total number of participants in the current study to 435. The mean age of the sample was 49.6 years (*SD* = 13.4) with a majority being female (88.4%) and having completed at least a standard college degree (69.1%).

##### Japan sample

Participants were recruited from physician clinics specializing in ME/CFS and the ME Japan association^2^. Among the 129 possible participants, five were excluded due to incomplete data. The current study included 124 participants which were 78.2% female with a mean age of 46.1 years (*SD* = 13.5), and 50.4% of them have completed at least a standard college degree.

##### Spain sample

Recruited by a specialist physician with experience diagnosing ME/CFS from a tertiary referral center in Barcelona, Spain, participants were surveyed using Research Electronic Data Capture (REDCap), a tool used for online data collection (19). Participants were required to be at least 18 years of age to be eligible. There were 232 participants and 50 were excluded due to incomplete data. The sample in the current study included 182 participants with a mean age of 50.4 years (*SD* = 8.7). Most of the sample was female (87.2%) and less than a quarter (14.8%) completed at least a standard college degree.

##### Amsterdam sample

Participants were recruited from a group of individuals with a physician diagnosis of ME/CFS, and the patients had been referred to an outpatient clinic in the Netherlands (the CFS Medical Center in Amsterdam) specializing in ME/CFS. There was a total of 364 participants. After excluding 8 participants due to incomplete date, 356 participants were included in the current study. Less than half the sample (42.1%) obtained at least a standard college degree. The sample had a mean age of 37.0 years (*SD* = 11.4) and 78.4% of the sample was female.

#### PASC sample

In August 2020, study recruitment information was posted on several social media sites to recruit those who self-reported not recovering from COVID-19. Participants were asked to describe current symptoms (20). The sample size was 299, and the average age was 45.1 (*SD* = 12.88), with 90.3% being white and 81.6% being female.

### Measures

#### DePaul Symptom Questionnaire

Participants from both datasets completed the DePaul Symptom Questionnaire (DSQ-1) (21), a 54-item self-report measure of ME/CFS symptomatology, demographics, medical, occupational, and social history. The present study focused on 13 DSQ-1 neurocognitive domain items, which included many symptoms from the Canadian Consensus Criteria (14) ME/CFS case definition. On a five-point Likert scale, with 0 = none of the time, 1 = *a little of the time*, 2 = *about half the time*, 3 = *most of the time*, and 4 = *all of the time*, participants with ME/CFS were asked to rate the frequency of each symptom over the past 6 months. Similarly, using a five-point Likert scale with 0 = *symptom not present*, 1 = *mild*, 2 = *moderate*, 3 = *severe*, and 4 = *very severe*, participants were asked to rate the severity of each symptom over the past 6 months. In contrast, those in the PASC group were asked to rate the DSQ-1 symptoms as they were experienced during the first 2 weeks of their illness and at the current time point (the latter time point was used in the analyses). Both frequency and severity scores were standardized to a 100-point scale and the frequency and severity scores for each symptom were averaged to create one composite score per symptom. The DSQ-1 has demonstrated strong reliability and validity (22) as well as the ability to accurately differentiate individuals with ME/CFS from individuals with Multiple Sclerosis and Post-Polio Syndrome (23, 24).

##### Impairment

In the DSQ-1, there is one item that measures patients’ level of impairment by answering a 7-point Likert scale item that assesses the severity of participants’ impairment. This 7-point Likert scale was re-coded into two levels: severe impairment (e.g., bedbound or homebound) and moderate (e.g., able to work part time and leave the house but did not have energy for other activities) impairment.

#### Statistical procedure

##### Methods for replacing missing values

Participants were removed from analyses in the current study if they were missing more than 10% of items from the DSQ-1. Participants could have missing data for either the severity, the frequency, or for both dimensions of a symptom. The missing values of the remaining participants were replaced with a method dependent on the nature of the missing value. For further details on how the missing values were replaced, please see Conroy, Islam, and Jason (25).

##### Factor analysis

IBM SPSS Statistics version 25 was used for all analyses (IBM Corps, 2017). Using the 100-point symptom composite scores, an exploratory factor analysis (EFA) was performed. A Promax rotation (kappa = 4) was used to allow the factors to correlate, and the principal axis factoring method was selected to determine the maximum amount of common variance between the factors. To determine the appropriate number of factors to retain, we constructed a parallel analysis using 5,000 iterations of our data using permutations and compared the changes in eigenvalues across consecutive factors to those of our actual data. If the respective eigenvalue exceeded that of the random data based on a 95% confidence interval, factors from the actual data were retained. Among the retained factors, those appearing before the inflection point of the scree plot were assumed to be meaningful. Symptoms that did not load onto any of the factors (rotated loading < 0.3) were dropped, and the analysis was repeated until all symptoms loaded onto a factor.

Lastly, we performed one-way ANOVAs to compare severe versus moderate impairment with the scores of factors from the exploratory factor analysis.

## Results

### Factor analysis

In the ME/CFS sample, the Kaiser-Meyer-Olkin (KMO) measure of sampling adequacy (0.94) and Bartlett’s Test of Sphericity [χ^2^_(78)_ = 16023.54, *p* < 0.001] supported the practicality of conducting an EFA. Principal axis factoring analysis conducted on the 13 DSQ-1 neurocognitive domain items yielded a two-factor solution (see Table 2). The first factor had an eigenvalue of 6.06 that accounted for 47% of the common variance, with factor loadings ranging from 0.60 to 0.89; the second factor had an eigenvalue of 0.87 that accounted for 7% of the common variance, with factor loadings ranging from 0.34 to 0.84. While the second factor explained a small amount of common variance, this factor was deemed meaningful and thus was retained. The correlation between the two factors was 0.65. Within the second factor, loss of depth perception and twitching had lower factor loadings so, a secondary EFA was conducted excluding these two items. The removal of the two items did not change or improve the overall factor structure or conceptualization of the analyses so they were included in the final results.

**Table 2:** Factor loadings of neurocognitive domain items in participants with ME/CFS.

Neurocognitive domain items	Factor 1	Factor 2
Absent-mindedness or forgetfulness	0.893	
Problems remembering things	0.865	
Slowness of thought	0.808	
Difficulty understanding things	0.806	
Difficulty finding the right word to say or expressing thoughts	0.788	
Difficulty paying attention for a long period of time	0.718	
Only able to focus on one thing at a time	0.664	
Unable to focus vision and/or attention	0.602	
Sensitivity to light		0.844
Sensitivity to noise		0.741
Sensitivity to smells		0.567
Twitching		0.342
Loss of depth perception		0.340

N = 2,313.

In the PASC sample, the KMO measure of sampling adequacy (0.94) and Bartlett’s Test of Sphericity [χ^2^_(78)_ = 3103.85, *p* < 0.001] supported the practicality of conducting an EFA. Principal axis factoring analysis conducted on the same 13 DSQ neurocognitive domain items yielded a two-factor solution (see Table 3). The first factor had an eigenvalue of 7.41 that accounted for 56% of the common variance, with factor loadings ranging from 0.69 to 0.95; the second factor had an eigenvalue of 0.91 that accounted for 7% of the common variance, with factor loadings ranging from 0.47 to 0.90. While the second factor explained a small amount of common variance, this factor was deemed meaningful and thus was retained. The correlation between the two factors was 0.71.

**Table 3:** Factor loadings of neurocognitive domain items in participants with PASC.

Neurocognitive domain items	Factor 1	Factor 2
Absent-mindedness or forgetfulness	0.945	
Slowness of thought	0.908	
Problems remembering things	0.905	
Difficulty paying attention for a long period of time	0.859	
Difficulty understanding things	0.842	
Difficulty finding the right word to say or expressing thoughts	0.840	
Only able to focus on one thing at a time	0.838	
Unable to focus vision and/or attention	0.687	
Sensitivity to noise		0.896
Sensitivity to light		0.811
Twitching		0.609
Loss of depth perception		0.495
Sensitivity to smells		0.469

*N* = 299.

### ANOVA

In the ME/CFS sample, we examined whether those with more functional impairment had worse scores on the two neurocognitive factors than those with less impairment (see Table 4). There was a statistically significant difference between the two levels of impairment in Factor 1 (impairment of concentration and memory) scores, [*F*(1, 2,204) = 124.47, *p* < 0.01]. For Factor 2 (sensory and perceptual disturbances), the severe and moderately impaired groups were also significantly different [*F*(1, 2,204) = 165.67, *p* < 0.01]. Similarly, in the PASC sample, both severe and moderate levels of impairment were statistically different for both factors, with the impairment of concentration and memory factor [*F*(1, 287) = 54.01, *p* < 0.01] and the sensory and perceptual disturbances factor [*F*(1, 287) = 54.24, *p* < 0.01].

**Table 4:** Means and standard deviations of factor scores and levels of impairment.

ME/CFS	Severe impairment	Mild/Moderate impairment	*p* ≤ 0.01
	Mean (*SD*)	Mean (*SD*)	
Impairment of concentration and memory	0.14 (0.93)	−0.36 (0.97)	**
Sensory and perceptual disturbances	0.15 (0.87)	−0.38 (0.88)	**

PASC	Severe impairment	Mild/Moderate impairment	*p* ≤ 0.01
	Mean (*SD*)	Mean (*SD*)	
Impairment of concentration and memory	0.41 (0.86)	−0.36 (0.91)	**
Sensory and perceptual disturbances	0.37 (0.91)	−0.35 (0.76)	**

## Discussion

In both samples, two similar factors emerged. Factor 1 involved neurocognitive symptoms specific to impairments with executive dysfunction, memory, and concentration. Factor 2 contained symptoms more specific to hypersensitivities and sensory and perceptual disturbances. In both ME/CFS and PASC samples, individuals who reported more severe impairment in their physical functioning had worse neurocognitive symptoms for both concentration/memory and sensory/perceptual factors. This finding provides some validation for the notion that these neurocognitive symptoms are related to individuals’ functional status.

One possible explanation of the neurocognitive symptoms is Menon’s (26) Triple Network Model, which is made up of the Central Executive Network, the Salience Network, and the Default Mode Network. According to this model, aberrances in each of these brain interconnective networks influence the functioning of other related networks. Factor 1 symptoms are directly related to dysfunctions of the central executive network, which is responsible for executive function, working memory, and attention (27). Indeed, patients with ME/CFS have been shown to have a decrease in the central executive network and the salience network functional connectivity when compared to controls, implying aberrances within these networks (28). Dysfunction of the salience network in the brain, which is primarily responsible for the “switching” between default mode and central executive networks, may be one explanation for the presentation of these neurocognitive symptoms, and perhaps other symptom domains, in people with ME/CFS and PASC.

Factor 2 contains symptoms more specific to hypersensitivities involving sensory and perceptual disturbances, which have been found among patients with PASC (16) and ME/CFS (17). These symptoms may be due to an overactivation of the anterior insula within the salience network, leading to excessive sensitivity. This is likely related to the low excitability threshold of the anterior insula, with excessive stimuli leading to over mobilization of reactions in non-threatening situations (29, 30). Therefore, the symptoms involving sensitivities to lights, noises, and smells could be the result of anterior insula overactivation within the salience network. In support of these notions, Manca et al. (28) found increased activation of the salience network in patients with ME/CFS. Hypervigilance caused by chronic orthostatic intolerance can lead to stimulus uptake in the sensory organs, while stimulus processing in corresponding brain regions may be impaired because of a reduced cerebral blood flow that is often seen in patients with ME/CFS and PASC (31).

There are several limitations in the current study. The sample sizes were not equal with considerably more patients in the ME/CFS sample. In addition, the findings are based on self-report measures and diagnoses of ME/CFS and PASC, so patients’ diagnoses were not confirmed by medical evaluations. Furthermore, neurocognitive impairment was assessed by a self-report inventory but not by biological measures. Although there is some concurrent validity based on measures of functional status, there is a need to relate these two neurocognitive domains to a wider battery of biobehavioral measures. It is of note that there are several medical conditions comorbid with ME/CFS that have also been related to abnormalities in the salience network (27, 29). While such comorbidities are present in individuals with ME/CFS and Long-COVID, distinguishing the specific contribution of each condition to the observed neurocognitive impairments and salience network abnormalities is complex and beyond the scope of the current analysis. Future research should further examine how comorbid conditions may interact with neurocognitive dysfunction and salience network abnormalities across ME/CFS and Long-COVID populations.

Classifying the mechanisms behind the complex neurocognitive symptomology of ME/CFS and PASC could provide physicians and patients with a better understanding of the diseases’ symptoms. Our findings suggest that the neural mechanisms behind the neurocognitive symptoms of post-viral illnesses might involve more areas including those involving sensory overload.

## Data Availability

The raw data supporting the conclusions of this article will be made available by the authors, without undue reservation.

## Footnotes

^1^ https://solvecfs.org

^2^ https://mecfsjapan.com
